# Self-Assembled Epitaxial Cathode–Electrolyte Nanocomposites for 3D Microbatteries

**DOI:** 10.1021/acsami.2c09474

**Published:** 2022-09-06

**Authors:** Daniel M. Cunha, Nicolas Gauquelin, Rui Xia, Johan Verbeeck, Mark Huijben

**Affiliations:** † MESA+ Institute for Nanotechnology, University of Twente, 7500 AE Enschede, Netherlands; ‡ Electron Microscopy for Materials Science (EMAT), University of Antwerp, 2020 Antwerp, Belgium

**Keywords:** battery, thin film, nanocomposite, self-assembly, epitaxy

## Abstract

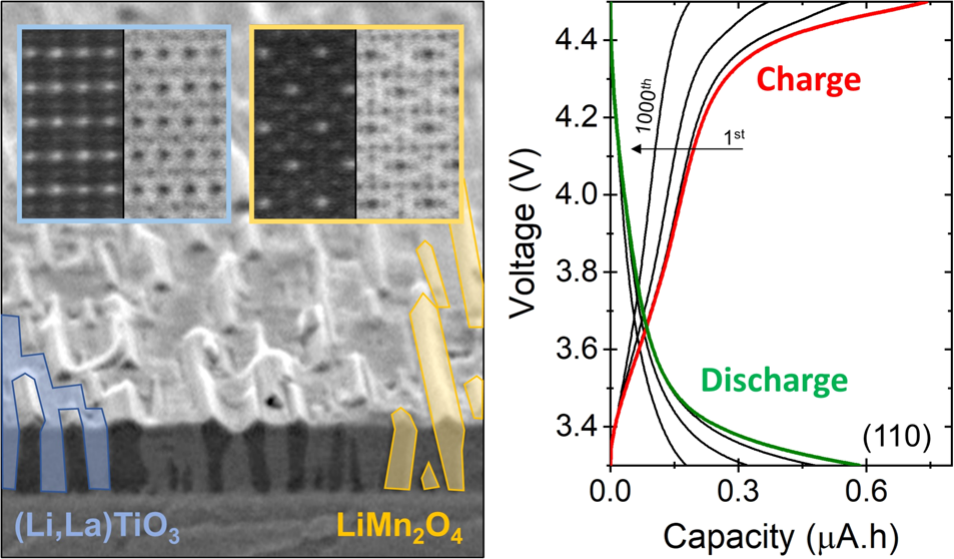

The downscaling of electronic devices requires rechargeable microbatteries with enhanced energy and power densities. Here, we evaluate self-assembled vertically aligned nanocomposite (VAN) thin films as a platform to create high-performance three-dimensional (3D) microelectrodes. This study focuses on controlling the VAN formation to enable interface engineering between the LiMn_2_O_4_ cathode and the (Li,La)TiO_3_ solid electrolyte. Electrochemical analysis in a half cell against lithium metal showed the absence of sharp redox peaks due to the confinement in the electrode pillars at the nanoscale. The (100)-oriented VAN thin films showed better rate capability and stability during extensive cycling due to the better alignment to the Li-diffusion channels. However, an enhanced pseudocapacitive contribution was observed for the increased total surface area within the (110)-oriented VAN thin films. These results demonstrate for the first time the electrochemical behavior of cathode–electrolyte VANs for lithium-ion 3D microbatteries while pointing out the importance of control over the vertical interfaces.

## Introduction

The continuous downscaling of electronic devices, such as medical implants, microsensors/actuators, wearable gadgets, self-powered integrated circuits, or microelectromechanical systems (MEMS), requires rechargeable microbatteries with enhanced energy and power densities.^1−3^ Commonly, microbatteries are structured as two-dimensional (2D), stacked thin-film geometries, where the layered fabrication facilitates industrial integration. However, 2D structures have limitations in size, power, and energy densities, which do not fully satisfy energy storage needs, raising the interest in three-dimensional (3D) microbatteries. Different layouts of 3D microelectrodes have been studied,^1−3^ providing a more extensive surface area between cathode, electrolyte, and anode, improving the current output of solid-state batteries. Although this ensures a giant step in power and energy density, the fabrication of such 3D batteries relies commonly on complex, multistep fabrication processes, which lack the control of the structural matching at the materials’ interfaces. Control of the interfaces is crucial in such devices as it enhances ionic and electronic transport, increasing energy and power densities. Therefore, the benefits of 3D microbatteries can be better exploited if a synthetic route provides the structure control of such systems down to the nanoscale, in combination with tunable crystal orientations of the individual materials and their shared interfaces.

Self-assembled vertically aligned nanocomposite (VAN) thin films formed by two immiscible oxides can exhibit unique properties not available in single-phase materials.^4−7^ The immiscibility of the two phases, *e.g*. perovskite–spinel combinations,^8−11^ forms the foundation of the self-assembly procedure resulting in highly ordered nanopillar/matrix structures. Such epitaxial VANs are self-assembled through physical vapor deposition, generally pulsed laser deposition (PLD), without sequential deposition, as is required for planar multilayer films. For epitaxially directed self-assembly growth, lattice mismatch, surface energy, and kinetics of the two film components and the substrate are crucial for realizing specific vertically aligned nanostructures within the films. Three-dimensional VANs offer advantages over 2D multilayers as their functionalities can be tailored by the strain- or charge-coupling at their vertical interfaces. However, the degree of coupling and the significance of nanostructures depend on the nanopillar–matrix morphologies, including domain patterns and interfacial properties. In VANs based on perovskite–spinel combinations, the underlying substrate crystal structure can control the VAN morphology and orientation.^8−11^ This intrinsic control in VAN thin films is possible due to the epitaxial matching to the single crystal substrate and the specific lowest energy surface for the individual perovskite and spinel phases, respectively (100) and (111) planes.

Although a range of epitaxial VANs has been studied in the last decade, lithium-based VANs for battery applications have remained primarily unexplored.^12^ Interestingly, two recent studies by Qi et al.^13^ and Cunha et al.^14^ demonstrated the unique potential of lithium-based VANs for achieving 3D solid-state batteries with enhanced energy storage performance. While Qi et al. incorporated metal Au nanopillars into Li_2_MnO_3_ cathode thin films to improve the electrical conductivity,^13^ we integrated the high-voltage LiMn_2_O_4_ (LMO) cathode with the promising solid-state electrolyte Li_3*x*_La_2/3–*x*_□_1/3–2*x*_TiO_3_ (LLTO).^14^ Good agreement was found between experiments and solid-on-solid kinetic Monte Carlo simulation modeling on the shape, size, and distribution of the LMO pillars within the LLTO matrix. However, the impact of the crystal orientation of the underlying substrate on the VAN formation and, consequently, the final electrochemical performance of the 3D cathode–electrolyte VAN films remained unexplored.

In this study, we focus on the control of the VAN formation to enable interface engineering between the LMO cathode and the LLTO electrolyte for achieving enhanced electrochemical performance in future 3D microbatteries. LMO is a spinel cathode material with a lattice parameter of about 8.25 Å, a 3D lithium-ion diffusion framework, and only 2% deformation during lithium insertion/extraction.^15^ It is an alternative for current commercial lithium-ion cathode materials due to its relatively high operating voltage (4.1 V *versus* Li/Li^+^) and comparable energy density (theoretically 148 mAh·g^–1^) combined with low cost and no direct environmental or safety hazards. The perovskite LLTO electrolyte material with a lattice parameter of about 3.90 Å exhibits high ionic conductivities at room temperature (∼10^–3^ S·cm^–1^) that is competitive with conventional liquid electrolytes.^16^

In epitaxially directed self-assembly of VAN films, one phase is crystallographically well-matched to the substrate such that it nucleates, grows epitaxially, and forms the host matrix. The second phase epitaxially aligns with the matrix phase and may or may not seed its growth on the substrate depending on surface energy considerations and the relative concentrations of the two components in the film. Therefore, different substrate orientations are expected to modify the arrangement of nanopillars and matrix and, consequently, affect the electrochemical performance of the 3D-structured cathode–electrolyte nanocomposite films. Here, the crystallographic orientation of the conducting Nb-doped SrTiO_3_ (Nb:STO) substrate is varied ((100) and (110)) to modify the arrangement of the VAN structure from, respectively, LMO nanopillars to nanoneedles in an LLTO matrix. The corresponding square-like and rooftop-like surface morphologies demonstrate the highly ordered nature of the LMO and LLTO single phases formed during self-assembly of the different epitaxial VAN architectures. Analysis of the electrochemical performance in a half cell against lithium metal showed apparent differences. Both VAN structures demonstrated high initial energy densities but different degradation behavior during extensive cycling.

## Experimental Section

The studied VAN films were prepared using PLD from a sintered 67% La_0.5_Li_0.5_TiO_3_ + 33% LiMn_2_O_4_ target (30 wt % excess Li) at an oxygen pressure of 0.2 mbar and a substrate temperature of 850 °C, positioned 50 mm from the target. A KrF excimer laser was used, operating at 248 nm, 8 Hz, and a laser energy fluence of 2.3 J·cm^–2^, resulting in a growth rate of ∼0.15 Å/pulse. The Nb-doped STO substrates (perovskite, *a* = 3.907 Å, 0.5 wt % Nb doping), with out-of-plane orientations of (100) and (110), were preannealed in a tube oven at 950 °C for 90 min in an oxygen flow of 150 L·h^–1^. After deposition, the films were cooled to room temperature at an oxygen pressure of 0.2 mbar at 10 °C·min^–1^. A 50 nm conducting SrRuO_3_ (SRO) layer was deposited as an intermediate layer to enhance the electrical transport between the LMO cathode and the Nb:STO substrate.^17,18^

The crystal structure, surface morphology, and thickness of the thin films were investigated by X-ray diffraction (PANalytical X’Pert PRO diffractometer with Cu Kα radiation and 1/32 slit, in steps of 0.002° and 8.7 s·step^–1^), atomic force microscopy (Bruker ICON Dimension Microscope on tapping mode in air with Bruker TESPA-V2 cantilevers), scanning electron microscopy (Zeiss Merlin HR-SEM), and high-resolution scanning transmission electron microscopy with electron energy loss spectroscopy imaging (Thermo Fisher Titan X-Ant-Em operated at 300 kV).

For electrochemical characterization, the VAN films were cycled galvanostatically against a lithium metal anode in a liquid electrolyte. The experiments were performed in an electrochemical EC-ref cell by EL-CELL combined with a glass fiber separator of 1 mm thickness, 0.6 mL electrolyte with 1 M LiPF_6_ in 1:1 ethylene carbonate:dimethyl carbonate (EC:DMC), and a lithium metal anode. The electrochemical measurements were performed at 22 °C using a BioLogic VMP-300 system in a two-electrode setup. The samples were cycled between 3.3 and 4.5 V with currents of 3 μA, corresponding to a C-rate of approximately 5C. A potentiostatic period of 5 minutes ensures complete charge or discharge before the next step.

## Results and Discussion

**Figure 1:**
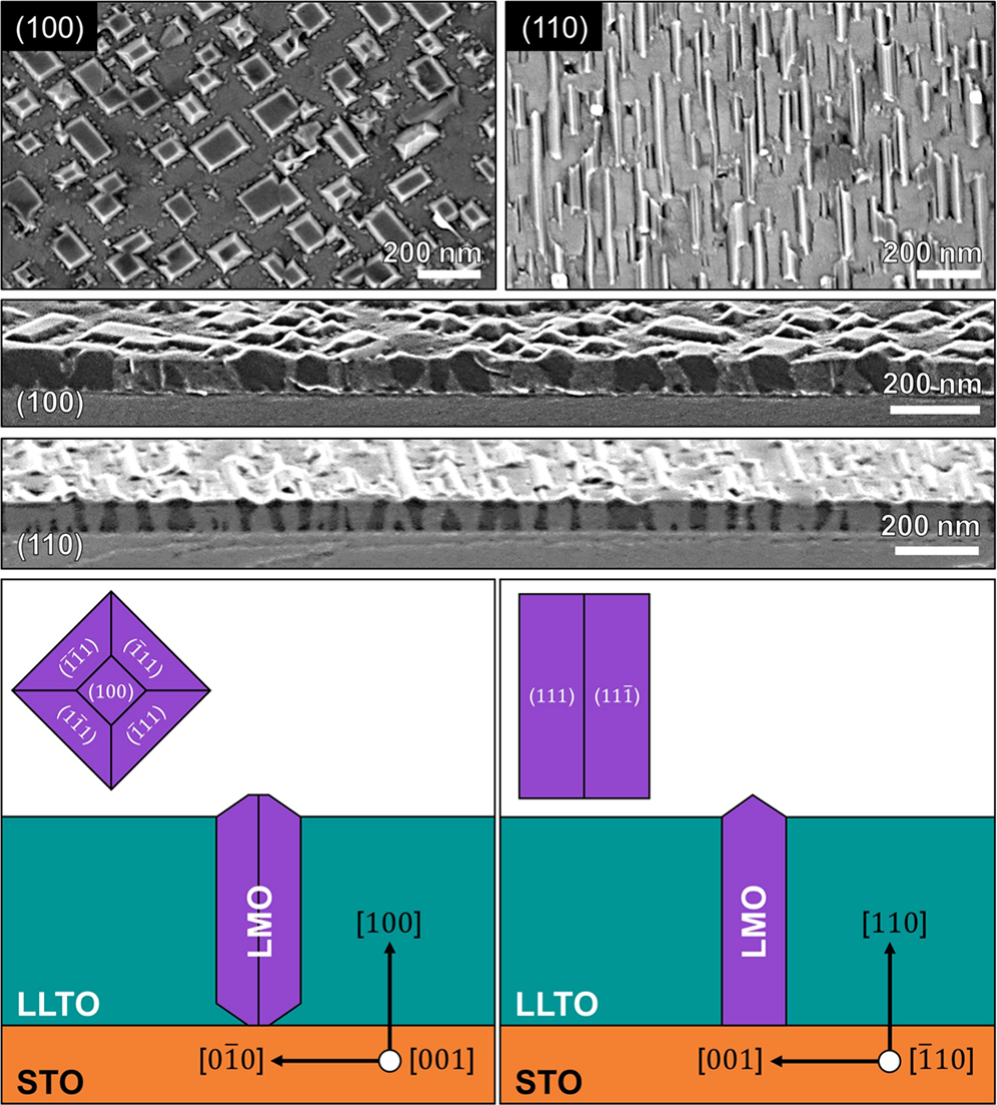
(Top) Top-view SEM images of the LMO–LLTO VAN films grown under the same deposition conditions on (100)- and (110)-oriented Nb:STO substrates. (Middle) Cross-sectional SEM analysis showing the formation of vertical nanopillars for different substrate orientations. (Bottom) Schematics illustrate the expected crystal facets for the square-like and rooftop-like surface morphologies for (100)- and (110)-oriented VAN films.

The distinct impact of the specific substrate crystal orientation on the achieved surface morphology of the VAN films can be observed in the scanning electron microscopy (SEM) analysis in Figure 1, showing square- and rooftop-like morphologies for, respectively, the (100)- and (110)-oriented Nb:STO substrates. The cross-sectional SEM images show the formation of vertical nanopillars within a surrounding matrix for both orientations with strong color contrast, indicating electronically conducting nanopillars (LMO) within an electronically insulating matrix (LLTO). The dimensions of the square-like nanopillars in the (100)-oriented VAN film are 111 ± 18 × 113 ± 23 nm^2^ (length × width), while the rooftop-like nanopillars in the (110)-oriented VAN film exhibit dimensions of 247 ± 74 × 49 ± 7 nm^2^. For a thickness of 110 nm, these dimensions correspond to a total LMO surface area 1.3 times higher for the (110) VAN structures over the (100), indicating that it is possible to tailor the pillar/matrix arrangements for Li-containing materials as reported for other systems.^9,19,20^ The square-like LMO nanopillars in the (100)-oriented VAN film show significant height differences at the surface (RMS = ∼16 nm), which is in good agreement with previously observed spinel structures in a VAN film.^17^ All four sides of such pyramidal LMO spinel structure consist of ⟨111⟩ crystal facets with the presence of a ⟨100⟩ crystal facet at the truncated top of the pyramid. The rooftop-like LMO nanopillars in the (110)-oriented VAN film exhibit a lower surface roughness (RMS = ∼8 nm) and elongated ⟨111⟩ crystal facets. This is caused by the anisotropic nature of the (110) crystal plane, which favors the diffusion of atoms along the [1̅10] direction compared to the [001] direction.^21^

**Figure 2:**
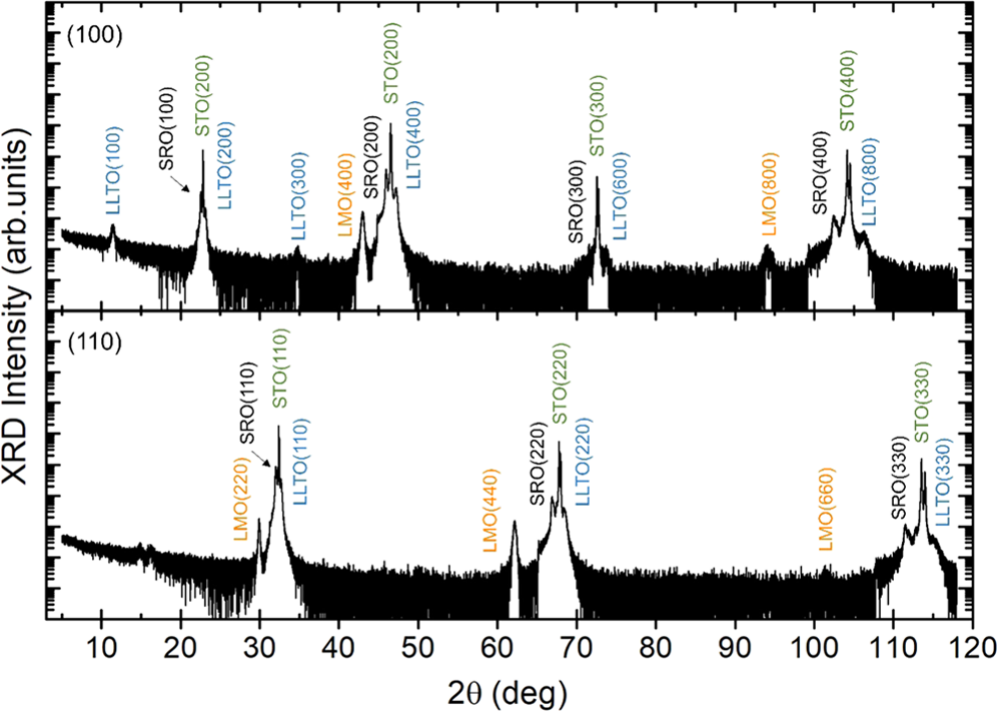
Out-of-plane XRD measurements of 110 nm epitaxial LMO–LLTO VAN films on 50 nm SrRuO_3_-coated Nb:STO substrates with different crystal orientations: (100), (110).

The successful self-assembly of the LMO and LLTO phases, without the formation of any impurity phases, was confirmed by X-ray diffraction (XRD) (Figure 2). The deposited LMO–LLTO VAN films exhibit coherent growth in which the out-of-plane crystal orientations of both LMO and LLTO structures are aligned with the out-of-plane Nb:STO substrate orientation, either (100) or (110). The LMO and LLTO peaks show highly crystalline, oriented spinel and perovskite structures, in agreement with previous studies of individual LMO or LLTO thin films grown on STO substrates.^17,22^ The results indicate that the achieved VAN structures stabilize the LMO phase at high temperatures of 850 °C, typically not achievable in single LMO thin films^17^ due to decomposition above 625 °C. This is expected to result from the out-of-plane strain induced by the surrounding LLTO perovskite matrix, similar to other material systems.^23,24^ The in-plane crystal orientations of the VAN films were studied by XRD φ-scans along the (013) direction for Nb:STO/SRO/LLTO (see Figure S1a) and indicate the in-plane alignment of the perovskite LLTO and SRO structures to the underlying Nb:STO perovskite substrate. The LLTO structure exhibits an out-of-plane lattice parameter of ∼3.85 Å, and it is strained in-plane to the STO (see Figure S1b) with a lattice parameter of 3.907 Å.^25^ This results in a unit cell volume of about 58.8 Å^3^, indicating an electrolyte composition of Li_0.36_La_0.55_TiO_3_.^26,27^ The relaxed LMO structure, as determined by RSM (Figure S1c), exhibits an out-of-plane lattice parameter of 8.305 Å, which is larger than the bulk LMO of 8.25 Å,^28^ suggesting a high lithiation state (Li*_x_*Mn_2_O_4_, with *x* > 1).^29,30^

**Figure 3:**
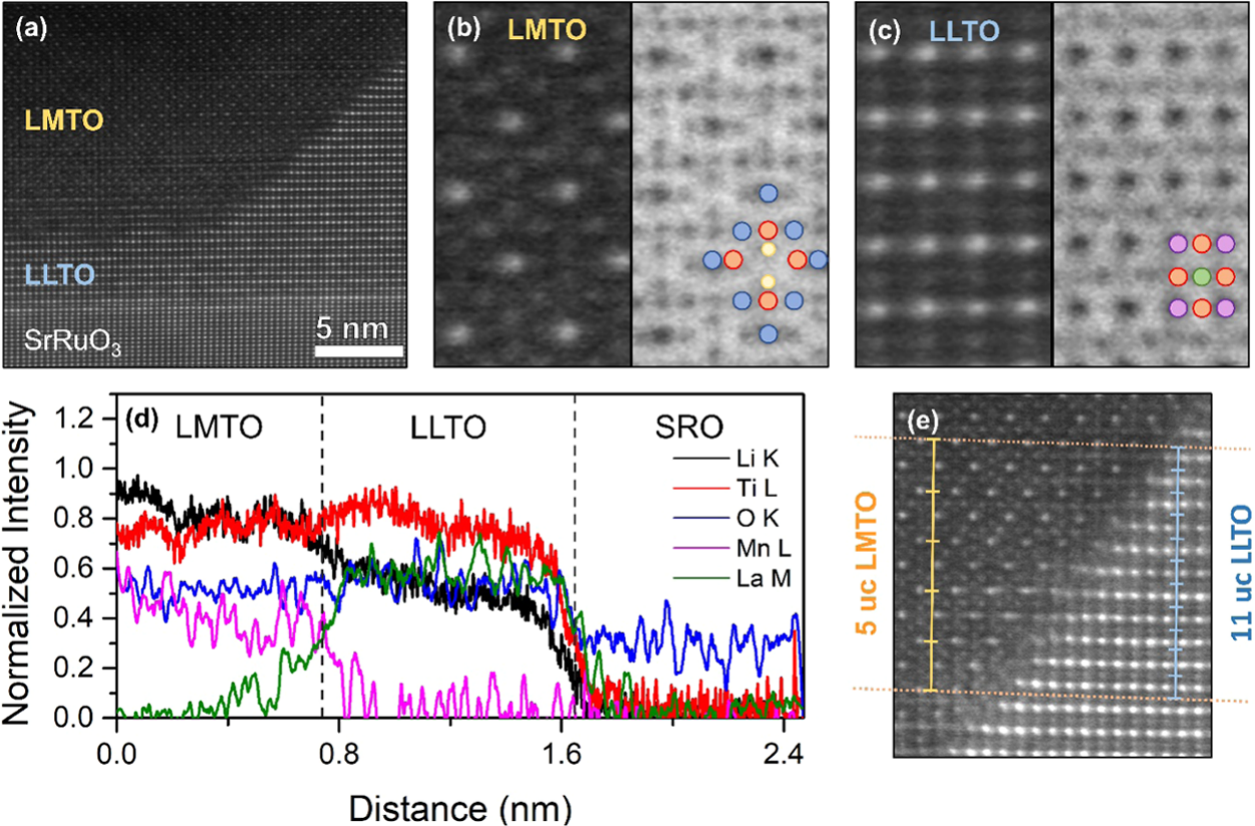
(a) STEM cross-sectional analysis of the (100)-oriented LMTO–LLTO VAN thin film, highlighting the (inverse) pyramidal morphology at the bottom of the LMTO nanopillar in contact with the LLTO matrix and SRO intermediate layer. (b, c) Detailed high-resolution STEM analysis (left) with the respective ABF image (right) of the atomic structural arrangement within the LMTO nanopillars and LLTO matrix. The colored spheres in the schematic represent (blue) Mn/Ti, (red) O, (purple) La, (green) Ti, and (yellow) Li atoms. (d) Elemental EELS analysis of the normalized intensity for the different elements across interfaces between LMTO, LLTO, and SRO. (e) Detailed STEM analysis of in-plane alignment across the interface between LMTO and LLTO.

More detailed structural and compositional analysis was performed on the (100)-oriented VAN film through high-resolution scanning transmission electron microscopy (HR-STEM) and electron energy loss spectroscopy (EELS) imaging, shown in Figure 3. Both perovskite LLTO matrix and spinel LMO nanopillar structures are observable in Figure 3a on top of the perovskite SRO buffer layer. However, similar to the top of the LMO nanopillars at the VAN surface, the bottom of the LMO nanopillar also exhibits an (inverse) pyramidal morphology, due to the low energy <111> crystal facets, in agreement with previous spinel–perovskite VAN films.^31^ This leads to a minimal contact area between the cathode LMO nanopillars and the conducting SRO layer, possibly limiting the electronic transport during electrochemical cycling in a battery device.

The LMO spinel structure within the nanopillars is shown in detail along the [100] crystal direction in Figure 3b. By applying annular bright-field (ABF) STEM imaging, the contrast has a low scaling rate with the atomic number allowing simultaneous imaging of light and heavy elements, resolving the lithium atoms within the structure.^32,33^ The ABF results demonstrate the fully lithiated state of the as-grown LMO structure within the VAN thin films as both locations for intercalating lithium atoms are filled. Similar ABF analysis of the LLTO matrix demonstrates the perovskite crystal structure. Furthermore, EELS analysis across the interface toward the SRO intermediate layer (see Figure S2), shown in Figure 3d, identifies a high concentration of Ti within the LMO nanopillar. Ti-doping of LMO (LMTO) has been studied previously as alternative cathode material, based on the enhanced structural stability, although with the cost of reducing the energy storage capacity and making the voltage plateaus less pronounced during battery cycling.^34,35^ Such Ti-doping of LMO is reported to cause an increase in the crystal structure lattice, in good agreement with the observed expansion up to 8.305 Å in the XRD results. Figure 3e depicts the in-plane alignment of the LMTO and LLTO crystal structures across the vertical interface. The difference between the spinel and perovskite crystal structures results in an epitaxial matching of about five LMTO unit cells (uc) with about 11 LLTO unit cells along the out-of-plane (100) direction.

**Figure 4:**
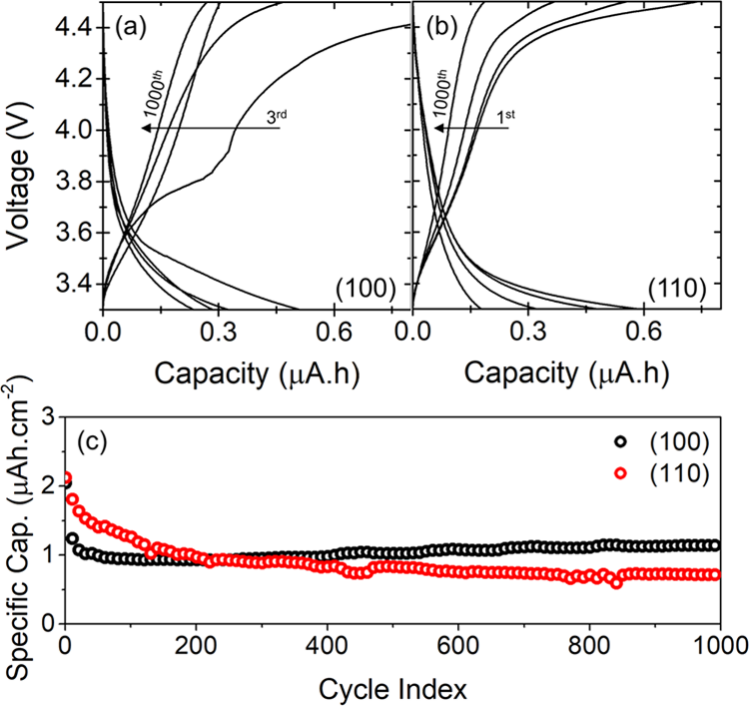
Cycling profile of LMTO–LLTO VAN thin films oriented in the (a) (100) and (b) (110) directions. (c) Long-term cycling stability test of (100)- and (110)-oriented VAN thin films.

The lithium intercalation characteristics were studied in electrochemical cells by galvanostatic charge–discharge analysis, shown in Figure 4. Considering the theoretical capacity of LMO (0.148 Ah·g^–1^), its density (4.28 g·cm^3^), and the LMO–LLTO target ratio of 0.33, the estimated capacity for the active LMO material within a 110 nm thick VAN thin film is 0.57 μAh (*i.e*. 2.3 μAh·cm^–2^ for 5 × 5 mm^2^ samples), in agreement with the observed capacity in Figure 4 for both oriented VAN thin films.

The typical double voltage plateaus of LMO at 4 V, for the redox reactions between Mn_2_O_4_–Li_0.5_Mn_2_O_4_–LiMn_2_O_4_, cannot be observed in the LMTO–LLTO VAN thin films’ charge–discharge profile. This behavior is possibly caused by the aforementioned Ti doping of the LMO pillars.^34,35^ Furthermore, reduction of the grain size of the active electrode material makes the plateaus less evident due to enhanced surface effects, as demonstrated by Okubo et al. for LiCoO_2_ with reduced grain sizes.^36^ The lateral sizes for pillars in the VAN thin films studied here are confined to about 100 nm for (100)-oriented VAN and about 50 nm for (110)-oriented VAN. Possible interphases between LMTO and LLTO within the VAN structures^37^ could cause even a further reduction of the active electrode material volume, although STEM results in Figure 3 suggest that the presence of such interphases is limited.

The charge–discharge behavior of the (100)- and (110)-oriented VAN thin films is quite similar. A slower voltage increase between 3.6 and 3.8 V can be observed in both films during the charging curves, indicating that the capacity of the nanocomposites is primarily from capacitive behavior. During the initial cycles, the capacity after charging is significantly higher than after discharging, leading to a low Coulombic efficiency (see Figure S3). This is caused by the high lithium level of the LMTO pillars after growth, as shown by STEM analysis in Figure 3, balanced during this initial cycling resulting in a constant Coulombic efficiency of about 90% after 50 cycles. The corresponding high initial discharge capacity after the first cycle of about 0.50 μAh and 0.53 μAh (*i*.*e*. 2 μAh·cm^–2^ and 2.1 μAh·cm^–2^ for the 5 × 5 mm^2^ samples), for, respectively, (100)- and (110)-oriented VAN thin films, changes significantly after continuous cycling. However, the behavior of the different VAN thin films during extensive cycling is very distinct. Although the (110)-oriented VAN exhibited the highest initial capacity of 2.1 μAh·cm^–2^, it displays a continuous capacity decrease down to 0.7 μAh·cm^–2^ after 1000 cycles. In contrast, the (100)-oriented VAN exhibited a sharp capacity drop from 2 to 0.9 μAh·cm^–2^ in the first few cycles, which changed into a slow capacity increase during further cycling. The capacity of the (100)-oriented VAN surpassed the (110)-oriented VAN after about 200 cycles and resulted in 1.1 μAh·cm^–2^ after 1000 cycles, equal to the capacity after the third cycle. These results are in good agreement with our previous study on single-layer crystalline LMO thin films exhibiting much higher rate capability and stability for the (100)-orientation compared to the (110)-orientation due to better alignment to the lithium diffusion channels.^17^

**Figure 5:**
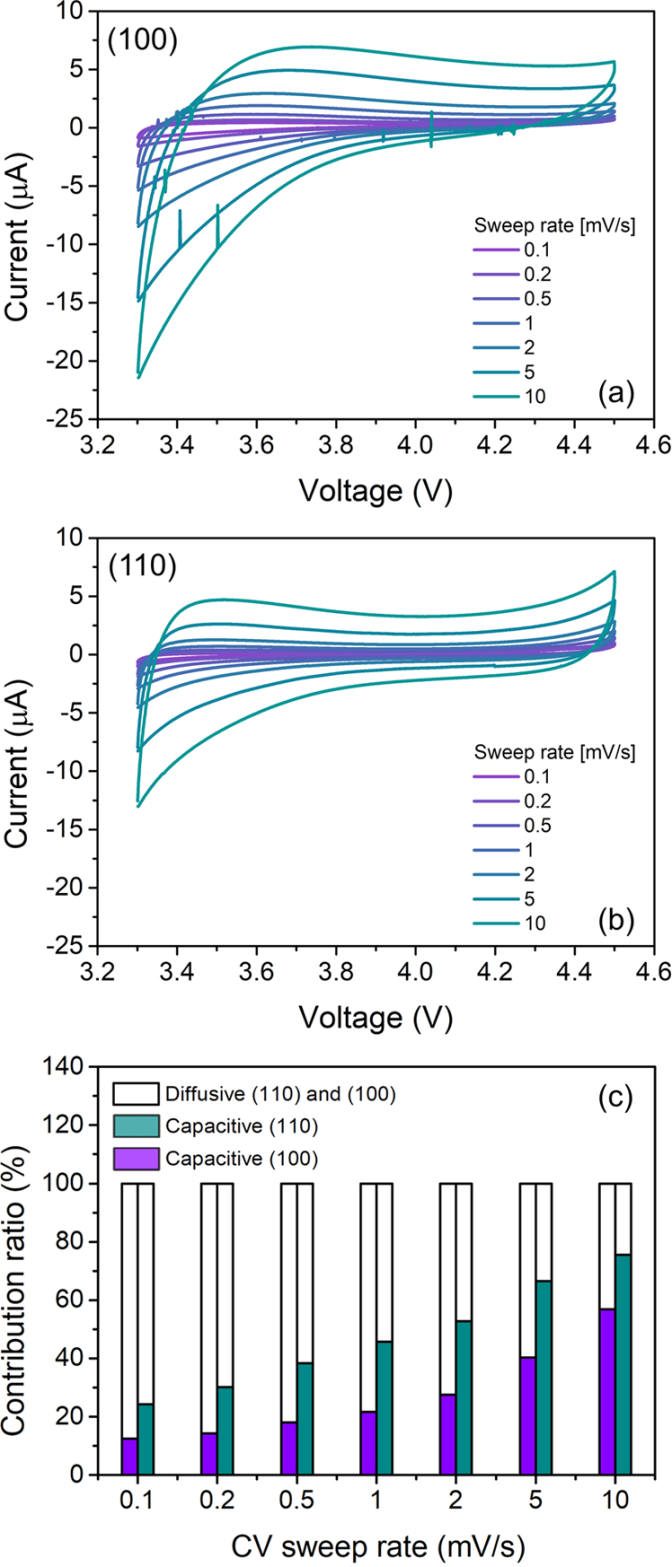
(a, b) Cyclic voltammetry analysis of LMTO–LLTO VAN thin films at different sweep rates from 0.1 to 10 mV·s^–1^. (c) Diffusive and pseudocapacitive contribution ratio for (100)- and (110)-oriented VAN thin films.

To obtain a detailed understanding of the electrochemical behavior of the LMTO–LLTO VAN thin films, different sweep rate cyclic voltammetry (CV) tests were performed between 3.3 and 4.5 V. As shown in Figure 5a,b, the CV curves of the (100)- and (110)-oriented VAN thin films do not show clear redox peaks, which agrees well with the absence of pronounced voltage plateaus during charge–discharge cycling (Figure 4). Subsequently, Dunn’s method was used to calculate the contributions of the surface and intercalation reactions to the specific lithiation process.^38^ The ratio between the surface chemical reactions (related to pseudocapacity) and the diffusion process (related to the intercalation capacity) was calculated for the variation of sweep rates between 0.1 and 10.0 mV·s^–1^.

Figure 5c shows the diffusive and pseudocapacitive contribution ratio for the (100)- and (110)-oriented LMTO–LLTO VAN thin films. The pseudocapacitive contribution of (100)-oriented VANs varies from 12.5% (0.1 mV·s^–1^) to 56.8% (10 mV·s^–1^) and for the (110)-oriented VANs from 24.2% (0.1 mV·s^–1^) to 75.6% (10 mV·s^–1^). These results show that all VAN thin films demonstrate the typical increase in pseudocapacitive contribution for higher CV sweep rates. However, this contribution is more dominant in the (110)-oriented VANs than in the (100)-oriented VANs. These results match the calculated increase in total LMTO surface area by 30% for the (110)-oriented VAN structures over the (100)-oriented VANs. When only the total top surface area of the LMTO nanopillars is considered, equal for both orientations, the lithium ions should have been transported directly from the liquid electrolyte into the LMTO electrodes. The presence of the same top surface area with mostly <111> crystal facets for (100)- and (110)-oriented VAN thin films suggests that the pseudocapacitive contribution should have been equal. Therefore, the observed experimental differences for the pseudocapacitive contributions should be caused by variations in lithiation processes at the vertical interfaces between the LMTO nanopillars and the surrounding LLTO electrolyte matrix. These results indicate that lithium transport through the LLTO electrolyte plays an important role, although further investigations are required.

## Conclusions

We have successfully demonstrated for the first time the electrochemical behavior of a cathode–electrolyte VAN structure as a promising direction for future 3D microbatteries. Control over the self-assembly process leads to well-defined nanopillar–matrix structures. The substrate orientation determines the morphology and the internal crystal orientation of the two involved phases. Electrochemical analysis in a half cell against lithium metal showed the absence of sharp redox peaks due to the confinement in the electrode pillars at the nanoscale. The (100)-oriented VAN thin films showed better rate capability and stability during extensive cycling than the (110)-oriented VANs, which is expected to be caused by the better alignment to the diffusion channels for optimal intercalation. Furthermore, a clear effect by an enhanced pseudocapacitive contribution could be observed for the increased total surface area within the (110)-oriented VAN thin films, as compared to (100)-oriented VANs, demonstrating the importance of control over the vertical interfaces between the LMTO nanopillars and the surrounding LLTO electrolyte matrix. This study shows the promising electrochemistry in such cathode–electrolyte VAN structures. Still, follow-up studies are required focused on solid electrolytes to determine the full potential of these vertically aligned nanostructures for their application in future 3D microbatteries.

## Supporting Information Available

In-plane XRD analysis and reciprocal space mapping of the epitaxial alignment within the VAN structures; STEM imaging of the location within the VAN structure for EELS analysis; and Coulombic efficiencies for electrochemical cycling of the VAN structures (PDF)
