# Supplementary material for: Self-Assembled Epitaxial Cathode–Electrolyte Nanocomposites for 3D Microbatteries
Source: ACS Appl Mater Interfaces. 2022 Sep 6;14(37):42208–14. doi: 10.1021/acsami.2c09474 (PMC9501919; doi:10.1021/acsami.2c09474)
Supplement: Supplementary file 1 — am2c09474_si_001.pdf [file am2c09474_si_001.pdf]

Supporting Information

Self-assembled epitaxial cathode-electrolyte nanocomposites for 3D micro-batteries

*Daniel M. Cunha[1], Nicolas Gauquelin[2], Rui Xia[1], Andre ten Elshof, Johan Verbeeck[2], Mark Huijben[1]**

[1] MESA+ Institute for Nanotechnology, University of Twente, 7500 AE Enschede, Netherlands

[2] Electron Microscopy for Materials Science (EMAT), University of Antwerp, 2020, Antwerp, Belgium

Corresponding Author

M. Huijben, m.huijben@utwente.nl

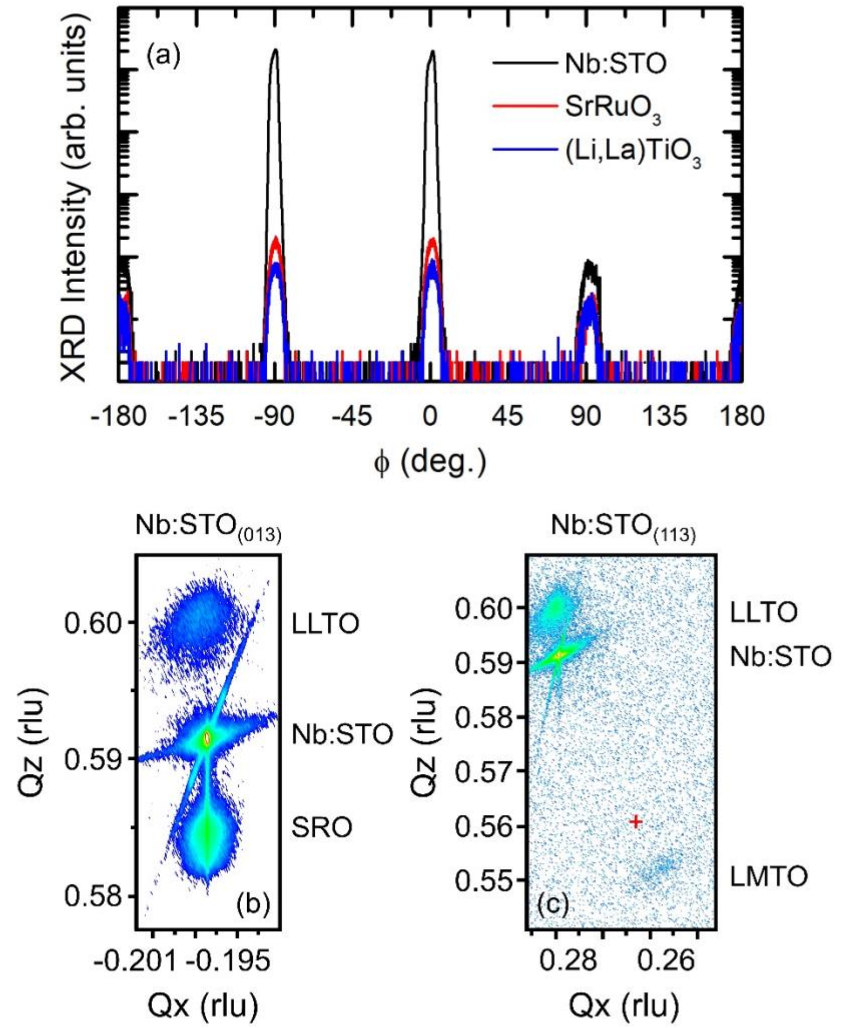

Figure S1. (a) In-plane XRD measurements of the LMO-LLTO VAN grown on STO(100), for the (013) plane. Peaks of the $Nb:SrTiO_3$, $SrRuO_3$ and $(Li,La)TiO_3$ are indicated respectively in black, red and blue. **(b)** Reciprocal space map graph obtained for the same film at the Nb:STO(013) plane highlighting the LLTO, Nb:STO and SRO peaks. **(c)** RMS graphs obtained at the STO (113), the red cross indicates the position expected for the relaxed bulk LMO.

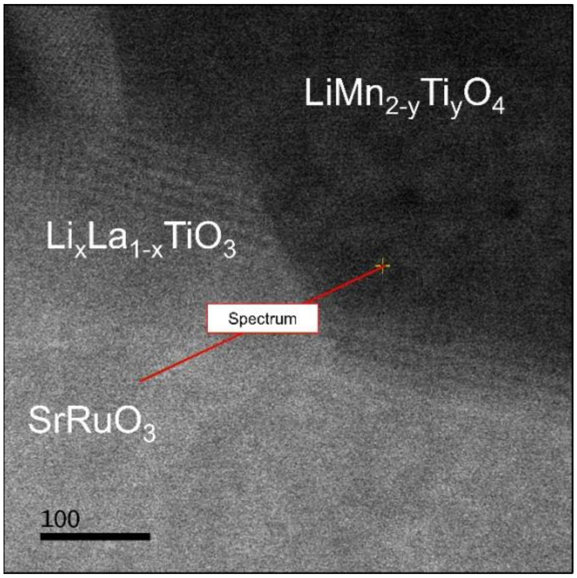

Figure S2. EELS analysis for the LMO-LLTO VAN grown on Nb:STO(100) showed in Figure 3d was performed on the profile highlighted in red.

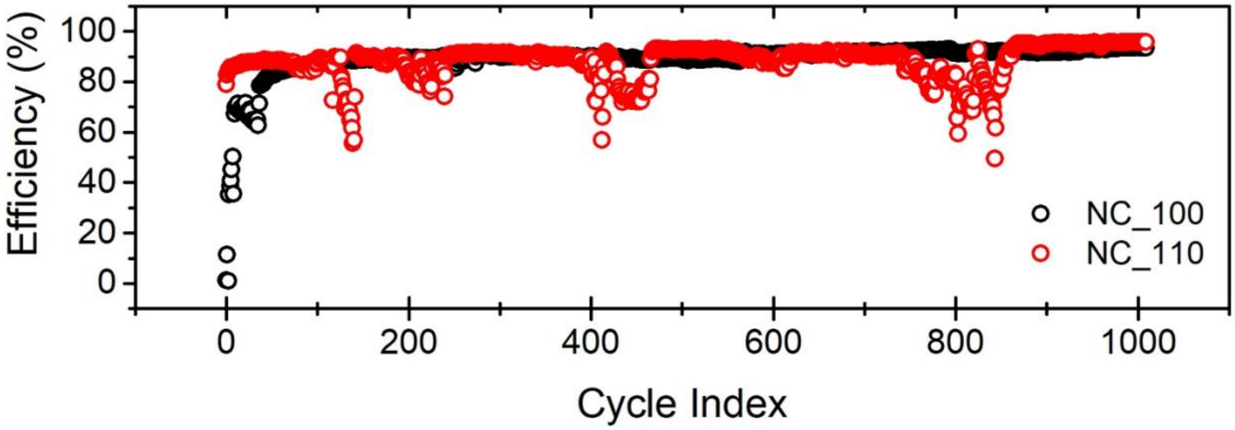

Figure S3. Coulombic efficiency given for a thousand cycles for the LMO-LLTO VAN grown on both crystallographic Nb:STO orientations.
